# Development and optimization of an ELISA method to detect *Toxoplasma gondii* oocyst infection in cats

**DOI:** 10.1007/s00436-025-08523-y

**Published:** 2025-07-07

**Authors:** Mingfeng He, Bufan Zhang, Shuai Han, Jiahui Qian, Zhengming He, Yulian Wei, Yanqin Zhou, Bang Shen, Rui Fang

**Affiliations:** State Key Laboratory of Agricultural Microbiology, College of Veterinary Medicine, Huazhong Agricultural University, Wuhan, 430070 Hubei Province People’s Republic of China

**Keywords:** *Toxoplasma gondii*, LEA880, IELISA, Oocyst infection, Feline toxoplasmosis

## Abstract

Toxoplasmosis caused by *Toxoplasma gondii *(*T. gondii*) is a zoonotic disease with great medical and veterinary significance. Felines, the definitive hosts of *T. gondii*, play a crucial role in the transmission of toxoplasmosis. The booming pet industry has led to more cats and cat-owning families, increasing the risk of toxoplasmosis transmission from animals to humans. Monitoring feline *T. gondii* infection accurately is crucial for reducing transmission risks. However, existing diagnostic methods focus on detecting whether cats are infected with *T. gondii* but fail to trace whether feline toxoplasmosis infections originate from oocysts or cysts. In this study, we assessed four late-stage development abundant proteins that were highly expressed specifically in sporulated oocysts to evaluate their specificity in binding to cat anti-*T. gondii*-oocyst serum. The LEA880 protein can only specifically react with cat anti-*T. gondii*-oocyst positive serum, but not with cat anti-*T. gondii*-cyst positive serum or negative cat serum. The optimized indirect enzyme-linked immunosorbent assay (iELISA) method based on the LEA880 protein exhibits good specificity and sensitivity in detecting *T. gondii* oocyst infection in cats.

**Supplementary Information:**

The online version contains supplementary material available at 10.1007/s00436-025-08523-y.

## Background

*Toxoplasma gondii* (*T. gondii*)is an important zoonotic pathogen causing toxoplasmosis (McLeod et al. 2012; Dubey et al. 2012). Humans, other mammals, and bird species are the intermediate hosts of *T. gondii* (Smith 1995). Approximately 30% of the world’s population is infected with *T. gondii* (Moncada and Montoya 2012). Although most infected individuals do not show clinical symptoms, the dormant parasites in tissues, for instance, muscles and the brain, pose a huge safety hazard (Dubey 1998). Alarmingly, for pregnant women, neonates, and HIV patients, *T. gondii* exhibits extremely strong pathogenicity (Hegab and Al-Mutawa 2003). Parasites transmitted vertically from the mother to the neonate can lead to neonatal malformations and even death (Montoya and Liesenfeld 2004; Kieffer and Wallon 2013; Milne et al. 2023). In immunocompromised individuals, central nervous system disorders, eye diseases, and carditis are common pathological manifestations of toxoplasmosis (Holliman et al. 1991; Montoya and Liesenfeld 2004; Cunningham et al. 2015; Zhou et al. 2021; Kalogeropoulos et al. 2022; Goh et al. 2023). Furthermore, toxoplasmosis also poses a substantial threat to the aquaculture industry. *T. gondii-*induced abortions in sheep result in significant global economic losses for the sheep industry (Buxton et al. 2007; Dubey 2009a). Porcine toxoplasmosis is prone to be accompanied by other diseases, including porcine reproductive and respiratory syndrome virus (PRRSV), classical swine fever virus (CSFV), and porcine circovirus type 2 (PCV-2) (Klein et al. 2010; Wang et al. 2015), which affects swine production and pork quality. Consequently, toxoplasmosis should receive more attention.

As the definitive host of *T. gondii*, felines play a crucial role in the life cycle of the parasite and its transmission (Miller et al. 1972; Dubey 2009b). Studies have revealed that there is a unique deficiency in delta-6-saturase activity in the intestines of felines (Genova et al. 2019), which enables *T. gondii* to undergo sexual reproduction within its small intestinal epithelial cells (Frenkel 1970). Feline feces contain immature/unsporulated oocysts, which become mature/sporulated in the environment. Statistically, a cat can excrete millions of oocysts after being infected with *T. gondii* (Dubey 2005). Oocysts exhibit high resistance to environmental and chemical damage (Freppel et al. 2019) and become infectious after sporulation, contaminating water, food, soil, and so on. Therefore, given the particularity and importance of cats in the transmission of toxoplasmosis, feline toxoplasmosis should be given due attention.

Feline toxoplasmosis exhibits a worldwide distribution (Rahimi et al. 2015; Kim et al. 2017; Shoshi et al. 2021; Galal et al. 2022). Validated indirect enzyme-linked immunosorbent assay (iELISA) diagnostic antigens for feline toxoplasmosis include *T. gondii* soluble antigen (TSA), surface antigen 1 (SAG1), and microneme protein 17 A (MIC17A). TSA is the natural antigen from *T. gondii* tachyzoites (Mineo et al. 1993; Bastos et al. 2016) and was first applied in the diagnosis of toxoplasmosis. Given the potential biosafety risks and standardization limitations, TSA has been replaced by recombinant proteins or multi-antigen combinations. SAG1 is mainly expressed during the parasite’s tachyzoite stage and plays an important role in the early stage of invading host cells (Kasper et al. 1984; Grimwood and Smith 1996). In the previous studies, SAG1 has been widely used in the serological diagnosis of *T. gondii* infections in dogs and cats (Kimbita et al. 2001; Hosseininejad et al. 2009; Chong et al. 2011; Hosseininejad 2012). MIC17A is currently the best recombinant antigen for the diagnosis of feline toxoplasmosis and is mainly expressed during the parasite merozoite stage. The MIC17A-iELISA diagnostic method demonstrates a significantly higher positive detection rate compared to the approach relying on tachyzoite and bradyzoite antigens (such as SAG1 and GRA1). Moreover, its accuracy is fivefold higher than that of the commercial IDvet diagnostic kit (Chen et al. 2022). Cats are mainly infected postnatally with *T. gondii* through two forms: oocyst infection and cyst infection (Frenkel 1970), and the likelihood of tachyzoite infection is substantially low (Robert-Gangneux and Dardé 2012). However, current diagnostic technologies, both domestic and international, can only determine whether a cat is infected with *T. gondii*, and there is no detection technology capable of distinguishing the routes of infection. Consequently, developing a diagnostic method to identify the infection route of feline toxoplasmosis is of great significance for source prevention and control.

To develop an ELISA method for detecting *T. gondii* oocyst infection in cats, we attempted to screen out an antigen that can specifically recognize the cat anti-*T. gondii*-oocyst positive serum from the late-stage development abundant (LEA) protein family, which is specifically expressed during the oocyst stage of *T. gondii*. Given the above findings, we identified the LEA880 protein as the ideal diagnostic marker.

## Materials and methods

### Parasites and cells

*T. gondii* type II strain ME49 was kept in our laboratory. HFF cells (human foreskin fibroblasts, ATCC, USA) used for vitro culture of parasites were cultured in Dulbecco’s modified Eagle medium (DMEM, HY, China). The medium was supplemented with 10% fetal bovine serum (FBS, HY, China) and 0.1 mg/mL penicillin–streptomycin (HY, China).

### Prokaryotic expression of protein

The coding sequences of LEA proteins were cloned from the cDNA of the ME49 strain. The full-length sequences of LEA850, LEA870, and LEA880, as well as the truncated sequence of LEA860 (25-200aa), were respectively ligated to the pET-28a vector by the ClonExpress MultiS One Step Cloning Kit (Vazyme, China). Recombinant proteins with His tags were successfully expressed in *E. coli* BL21 (DE3) and purified by nickel column affinity chromatography (Cytiva, Sigma). Finally, the purified antigens were quantified using BCA protein concentration assay kit (HY, China).

### Western blot

Antigens were subjected to protein separation on a 12% sodium dodecyl sulfate–polyacrylamide gel electrophoresis (SDS-PAGE) gel and then transferred to a polyvinylidene fluoride (PVDF, Millipore, Sigma) membrane. The PVDF membrane was incubated separately with cat anti-*T. gondii*-oocyst positive serum, cat anti-*T. gondii*-cyst serum, and negative cat serum. Finally, it was detected by horseradish peroxidase (HRP) conjugated rabbit anti-cat IgG (Biolab, China).

### Animal infection with *T. gondii*

ICR female mice (7 weeks old) bred by the laboratory animal center of Huazhong Agricultural University were intraperitoneally infected with ME49 tachyzoites to induce *T. gondii* cysts. Subsequently, 400 cysts were fed to *T. gondii-*seronegative cats to obtain oocysts. A total of 1000 sporulated oocysts were orally administered to *T. gondii*-seronegative cats. Anti-*T. gondii* sera (against oocysts and cysts) were collected from the cats 30 days post-infection.

### Optimization of LEA880-iELISA

ELISA was performed as previously described (Song et al. 2021). The antigen was coated onto the microtiter ELISA plate (Biofil, China) and incubated overnight at 4 ℃. After blocking the plate with BSA, it was incubated with the cat serum to be tested. HRP-conjugated rabbit anti-cat IgG served as the secondary antibody. TMB buffer was added for color development, and the reaction was stopped with hydrofluoric acid (HF). Finally, the OD630 value of each well was measured by a microplate reader. The cutoff value was determined by calculating the OD630 of 20 negative cat sera (cutoff = mean + 3SD).

### Evaluation of LEA880-iELISA

The optimized LEA880-iELISA method was applied to detect antibodies against other feline pathogens including feline panleukopenia virus (FPV), feline herpesvirus (FHV), feline infectious peritonitis virus (FIPV), and feline coccidia, to evaluate its specificity. The lowest detection limit of LEA880-iELISA was determined by measuring the sample serum OD630/negative serum OD630 (S/N value) of serially diluted cat anti-*T. gondii*-oocysts positive sera. Finally, the LEA880-iELISA was applied in combination with the established diagnostic feline toxoplasmosis method to detect clinical samples.

### MIC17A-iELISA

The MIC17A-iELISA was a previously reported method for detecting feline toxoplasmosis (Chen et al. 2022). The microneme protein 17 (MIC17A) fused with a His-tag was expressed and purified. A 96-well plate was coated with MIC17A at a concentration of 2.25 µg/mL, and cat serum samples were diluted at a ratio of 1:100. The cutoff value of MIC17A-iELISA was 2.48.

### Statistical analysis

GraphPad Prism 9.5 was used to generate graphs.

## Results

### Screening and prokaryotic expression of LEA proteins

**Fig. 1:**
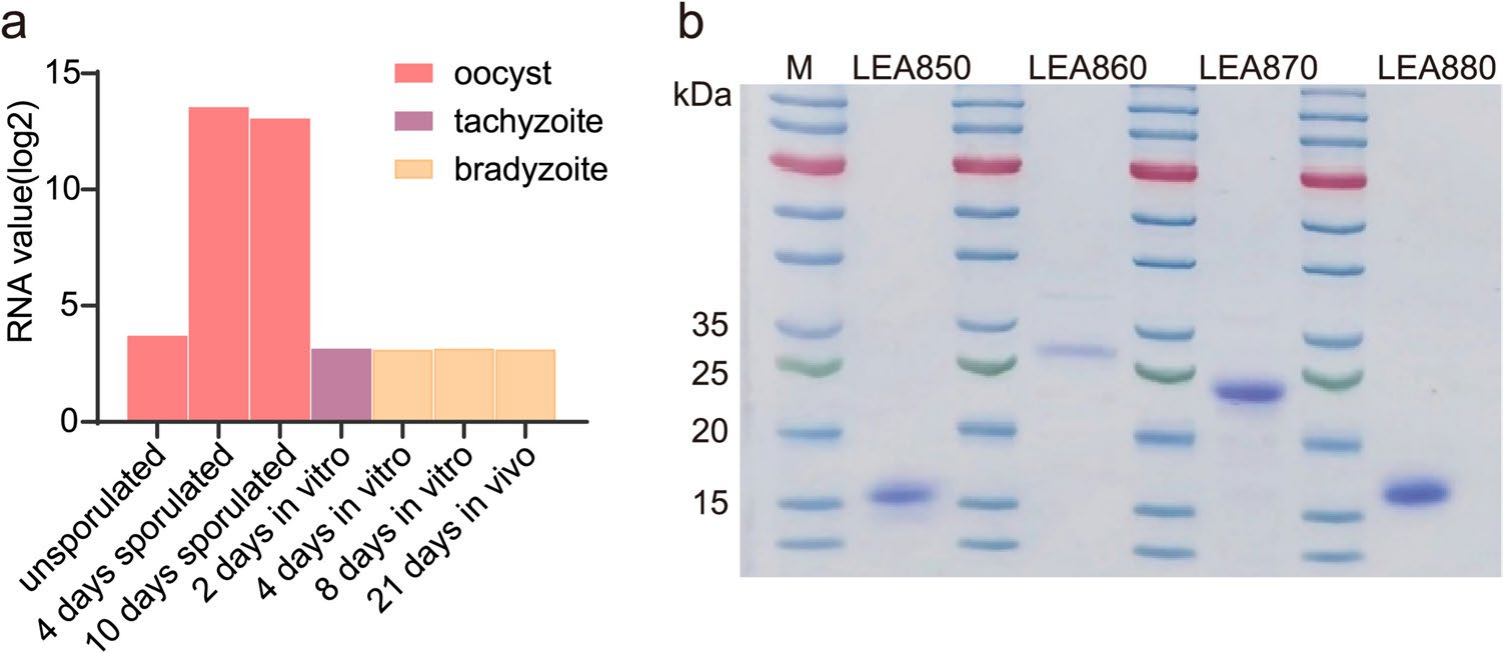
Developmental expression pattern diagram and recombinant expression of LEA proteins. **a** Oocyst, tachyzoite, and bradyzoite developmental expression pattern diagram of LEAs. Expression pattern diagram was simulated based on the LEA880 expression profiles. Data were extracted from TOXO.DB (oocyst, tachyzoite, and bradyzoite developmental expression profiles (M4) (Fritz et al. 2012a)). Expression level is shown as RMA normalized values (log base 2). **b** Separated the purified antigens on SDS-PAGE gel

To find a suitable antigen for tracing the infection route of feline toxoplasmosis, we screened four proteins identified as LEA proteins from the protozoan database TOXO.DB (https://toxodb.org/toxo/app). These proteins include LEA850 (TGME49_276850), LEA860 (TGME49_276860), LEA870 (TGME49_276870), and LEA880 (TGME49_276880). They exhibit identical developmental expression patterns in which genes are highly expressed specifically in sporulated oocysts (Fritz et al. 2012b) (Fig. 1a). Notably, the expression profiles of the four proteins are not entirely consistent. To further characterize LEAs, we recombinantly expressed them in *E. coli* and purified them by nickel column affinity chromatography. All antigens were subjected to protein separation on a 12% SDS-PAGE gel (Fig. 1b).

### LEA880 specifically reacts with the cat anti-*T. gondii*-oocyst positive serum

**Fig. 2:**
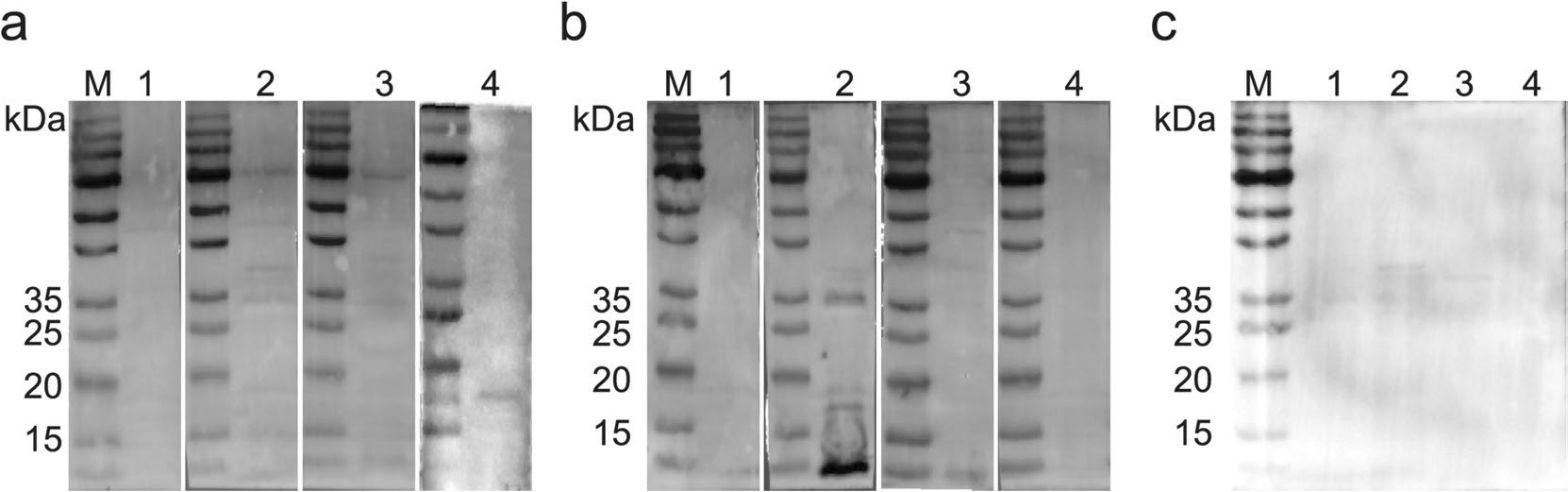
Western blot checking the reactivity of LEAs to cat sera. **a** Incubated with cat anti-*T. gondii*-oocysts positive serum. **b** Incubated with cat anti-*T. gondii*-cysts positive serum. **c** Incubated with negative cat serum. M: protein marker, Lane 1: LEA850, Lane 2: LEA860, Lane 3: LEA870, Lane 4: LEA880

To detect whether the LEAs can specifically react with the IgG antibodies in the cat anti-*T. gondii-*oocyst serum, we performed Western blot (WB) assays on the purified recombinant proteins with cat negative serum, cat anti-*T. gondii*-oocyst serum, and cat anti-*T. gondii-*cyst serum, respectively. The results showed that among the four proteins, only LEA880 specifically reacted with the cat anti-*T. gondii-*oocyst serum (Fig. 2a), but not with the cat anti-*T. gondii*-cyst serum (Fig. 2b) or the negative serum (Fig. 2c). The findings suggested that LEA880 holds potential for tracing the infection route of feline toxoplasmosis.

### Optimization of LEA880-iELISA procedure

**Fig. 3:**
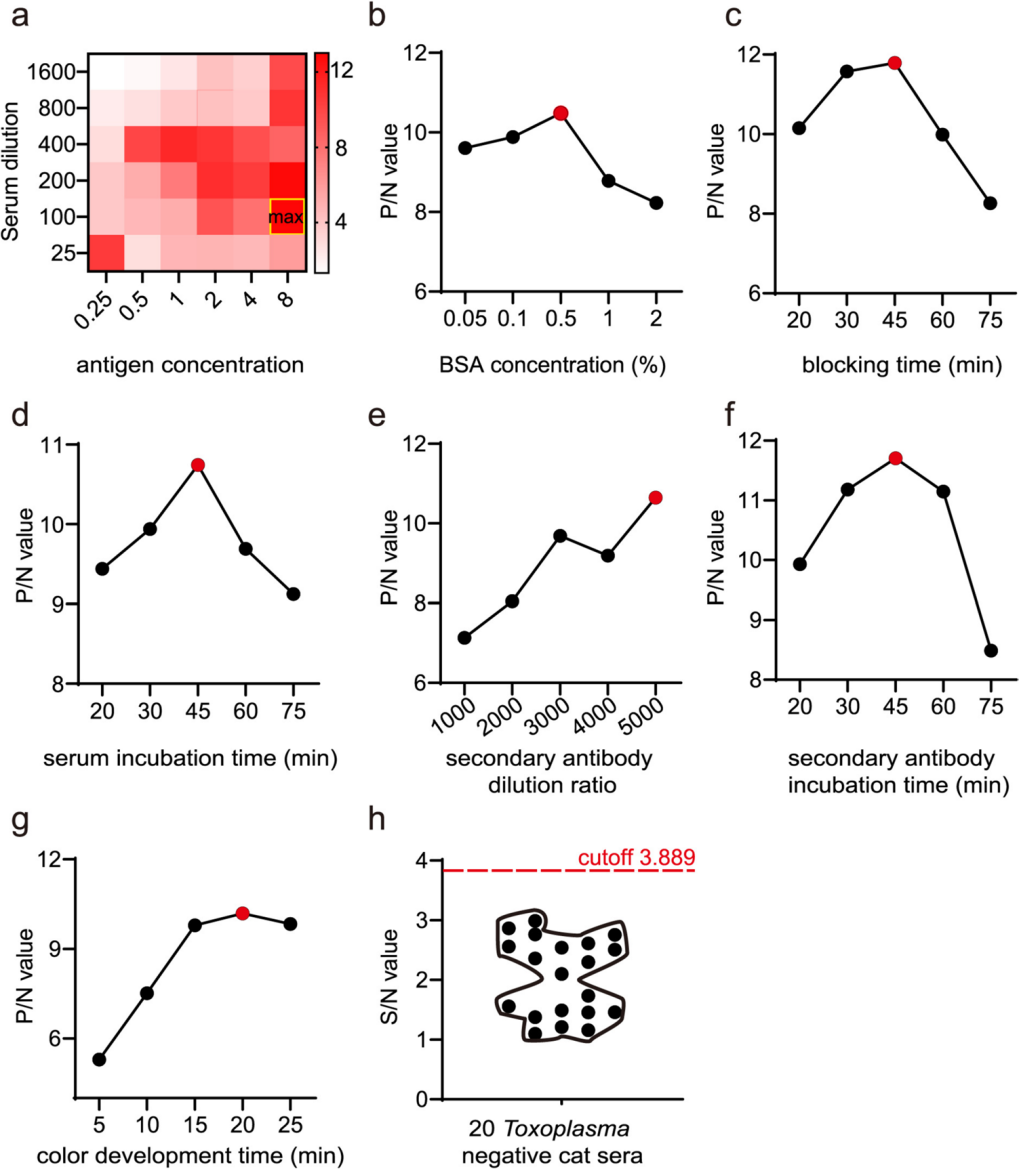
Optimization of LEA880-iELISA. **a** Antigen coating concentration and serum dilution ratio. **b** Blocking buffer concentration. **c** Blocking time. **d** Cat serum incubation time. **e** Secondary antibody dilution ratio. **f** Secondary antibody incubation time. **g** Color development time. **h** Cutoff value of cat anti*-T. gondii*-negative serum (cutoff = mean + 3SD)

To optimize the performance of LEA880-iELISA, we tested multiple conditions including the antigen coating concentration, serum dilution ratio, blocking buffer concentration, blocking time, serum incubation time, secondary antibody dilution ratio, secondary antibody incubation time, and color development time. The results of the cross-titration showed that the maximum positive serum OD630/negative serum OD630 (P/N value) was obtained when the coating concentration of LEA880 was 8 µg/mL, and the dilution ratio of cat serum was 1:100 (Fig. 3a). Selecting BSA as the blocking agent, the best effect was achieved with 0.5% BSA at 37 °C for 45 min (Fig. 3b, c). The optimal cat serum incubation time was determined to be 45 min (Fig. 3d). HRP-conjugated rabbit anti-cat IgG performed best when diluted at 1:5000 and incubated for 45 min at 37 °C (Fig. 3e, f). Finally, the optimal color development was achieved by incubating with TMB substrate for 20 min (Fig. 3g). Under the above optimal conditions, the cutoff value for LEA880-iELISA was 3.889 (Fig. 3h).

### Evaluation and application of LEA880-iELISA procedure

**Fig. 4:**
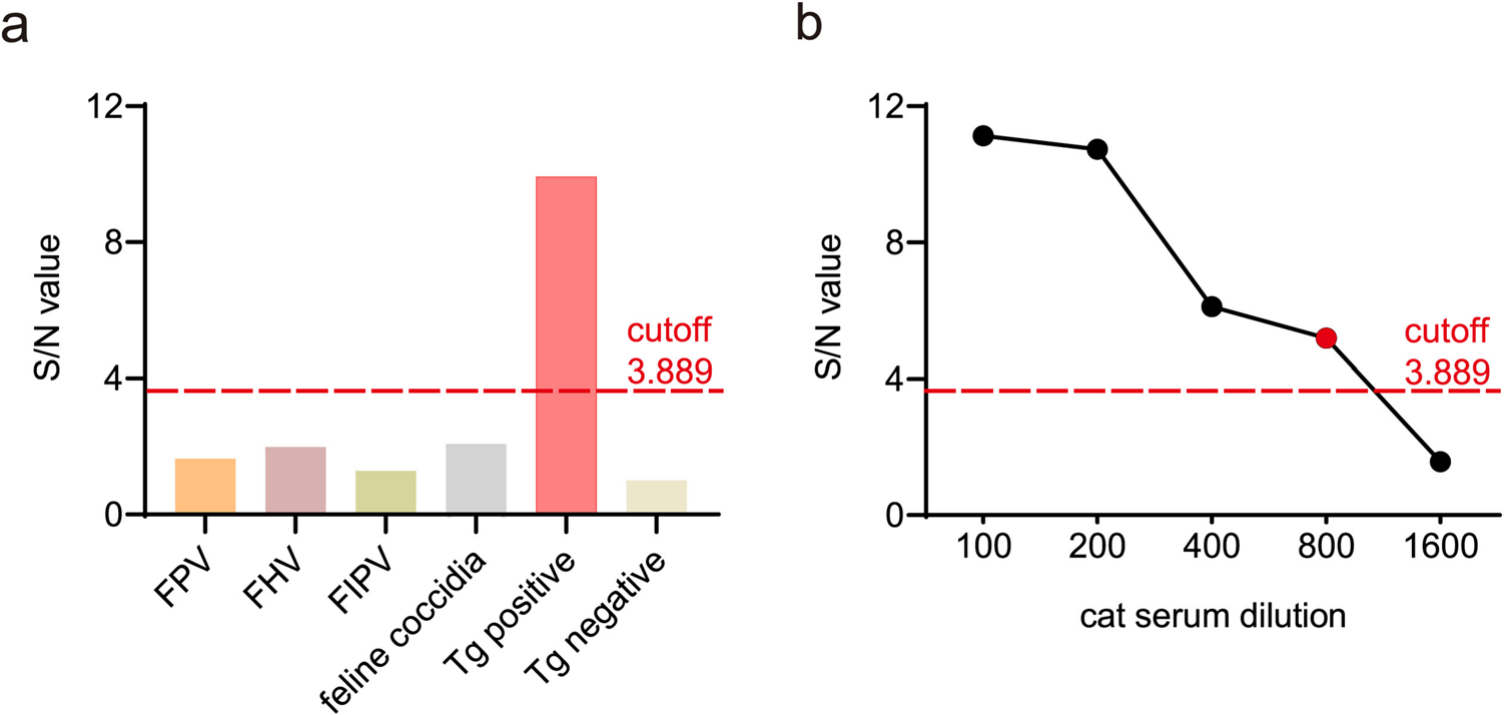
Evaluation of LEA880-iELISA. **a** Specificity of LEA880-iELISA was assessed by cross-reactivity with FPV, FHV, FIPV, and feline coccidia. **b** Lowest detection limit was determined by serial dilutions of cat anti-*T. gondii*-oocyst serum

To evaluate the feasibility of LEA880-iELISA procedure, we tested its specificity and sensitivity. Using the optimized procedure to detect the positive sera for FPV, FHV, FIPV, and feline coccidia, all the S/N value were negative (Fig. 4a), demonstrating that LEA880 does not cross-react with these pathogens and confirming the high specificity of the LEA880-iELISA. Meanwhile, to assess sensitivity, the cat anti-*T. gondii-*oocyst positive serum was serially diluted. The results demonstrated that the serum could still be detected as positive at a dilution of up to 1:800 (Fig. 4b), confirming the sufficient sensitivity of the method.

To evaluate the practical application of LEA880-iELISA in clinical samples, we tested 117 cat serum samples randomly collected from animal hospitals using both LEA880-iELISA and MIC17A-iELISA, which is recognized as the most effective method for detecting feline toxoplasmosis in the laboratory. The results demonstrated that all 15 sera positive for *T. gondii* oocyst infection detected by LEA880-iELISA were included within the 54 sera identified as positive by MIC17A-iELISA (Table 1). Furthermore, there were no instances where samples tested negative by MIC17A-iELISA but positive by LEA880-iELISA, indicating that the LEA880-iELISA method did not produce false positives in this sample detection.

**Table 1:** Samples detection by LEA880-iELISA and MIC17A-iELISA

		LEA880-iELISA results	
		Positive	Negative
MIC17A-iELISA results	Positive	15^a^	39
	Negative	0	63

^a^Number of serum samples in each category

## Discussion

Toxoplasmosis, as a zoonotic disease, presents a substantial public health risk. Felines, as the definitive hosts of *T. gondii*, play an important role in its complex life cycle. With economic development and increasing life stress, the number of cats as companion animals entering human households has increased steadily, which increases the risk of *T. gondii* transmission between animals and humans. Unfortunately, current diagnostic methods for toxoplasmosis only allow for a preliminary assessment of infection prevalence and distribution in epidemiological studies. There is still no reliable diagnostic method for tracing the infection route of feline toxoplasmosis.

Studies investigating toxoplasmosis infection routes in other animals have identified the LEA850 protein as a critical factor. LEA850 is an important member of the LEAs which are characterized by their specific expression during the oocyst stage of *T. gondii*. LEA850 showed specific reactivity to *T. gondii* oocyst–infected serum while maintaining the capacity to differentiate infection routes in humans, pigs, and mice (Hill et al. 2011). Researchers developed a diagnostic method with LEA850 to detect human serum IgG and salivary IgA, enabling epidemiological analysis of different infection modes of *T. gondii* in humans (Mangiavacchi et al. 2016). Therefore, in this study, we attempted to screen effective antigens from members of the LEA family that can be applied for the diagnosis and tracing of feline toxoplasmosis.

LEAs have been widely reported in plants and microorganisms, where they primarily function to resist various adverse external environmental changes (Tunnacliffe and Wise 2007). In *T. gondii*, LEAs are mainly expressed during the oocyst stage and highly upregulated in sporulated oocysts compared to unsporulated oocysts (Fritz et al. 2012a). The latest research demonstrated that combinatorial knocking of four *T. gondii* LEA family genes (LEA850, LEA860, LEA870, LEA880) significantly enhanced oocyst susceptibility to high salinity, drought, and cold conditions (Arranz-Solís et al. 2023). This functional conservation parallels the established protective role of LEA proteins across species.

In this study, four LEA proteins were successfully expressed in *E. coli.* Through WB assay, we found that LEA880 protein can only recognize the cat anti-*T. gondii*-oocyst positive serum, but not negative serum and cat anti-*T. gondii*-cyst positive serum, indicating its ability to trace the infection route in feline toxoplasmosis. This finding parallels the utility of LEA850 protein in identifying the infection pathway of porcine toxoplasmosis. Importantly, it highlights that proteins exclusively highly expressed in sporulated oocysts represents a far superior option for tracing *T. gondii* infection sources.

MIC17A-iELISA is currently a highly efficient method used for the diagnosis of feline toxoplasmosis, with a higher detection rate compared to TSA-iELISA. Our study combined LEA880-iELISA and MIC17A-iELISA for the detection of 117 cat serum samples. LEA880-iELISA detected 15 positive serum samples for oocyst infection, while MIC17A-iELISA detected 39 positive serum samples for *T. gondii* infection. The former was completely included in the latter’s results, while the detection of negative serum showed good consistency between the two methods. These results indicate that the highly expressed LEA880 protein during sporulation is a promising antigen for tracing the infection route of feline toxoplasmosis.

As the definitive host of *Toxoplasma gondii*, cats play a crucial role in the transmission of toxoplasmosis. Identifying the primary infection routes in cats is essential for tracing contamination sources and implementing effective control measures to reduce parasite transmission. In this study, 54 out of 117 feline serum samples tested positive for *T. gondii*-specific antibodies, with 39 cases (72.2%) attributed to cyst ingestion and 15 cases (27.8%) linked to oocyst exposure. The significant predominance of cyst-mediated infection highlights the importance of targeting meatborne transmission in prevention strategies. We recommend that cat owners adopt a “tissue cyst interruption” approach, including thorough cooking of meat, avoiding raw meat diets, and strict hygiene practices when handling raw meat, supplemented by daily litter box cleaning to mitigate secondary oocyst exposure. This integrated strategy effectively reduces the primary infection risk in cats, thereby minimizing potential transmission to humans. Proper pet management based on these findings can contribute to the source control of toxoplasmosis in both feline and human populations.

## Conclusion

We reported LEA880 as an ELISA diagnostic antigen for feline toxoplasmosis, which can recognize cat anti-*T. gondii*-oocyst positive serum but not cyst infection positive and negative serum. Compared with the established diagnostic method, LEA880-iELISA has good diagnostic performance in identifying the *T. gondii* oocyst infection, which is of great significance for the infection route tracing and comprehensive prevention and control of toxoplasmosis.

## Supplementary Information

Below is the link to the electronic supplementary material.

ESM 1 (ZIP 3.47 MB)

## Data Availability

No datasets were generated or analysed during the current study.
