# Supplementary figures and images for: Development and optimization of an ELISA method to detect Toxoplasma gondii oocyst infection in cats
Source: Parasitol Res. 2025 Jul 7;124(7):79. doi: 10.1007/s00436-025-08523-y (PMC12229958; doi:10.1007/s00436-025-08523-y)

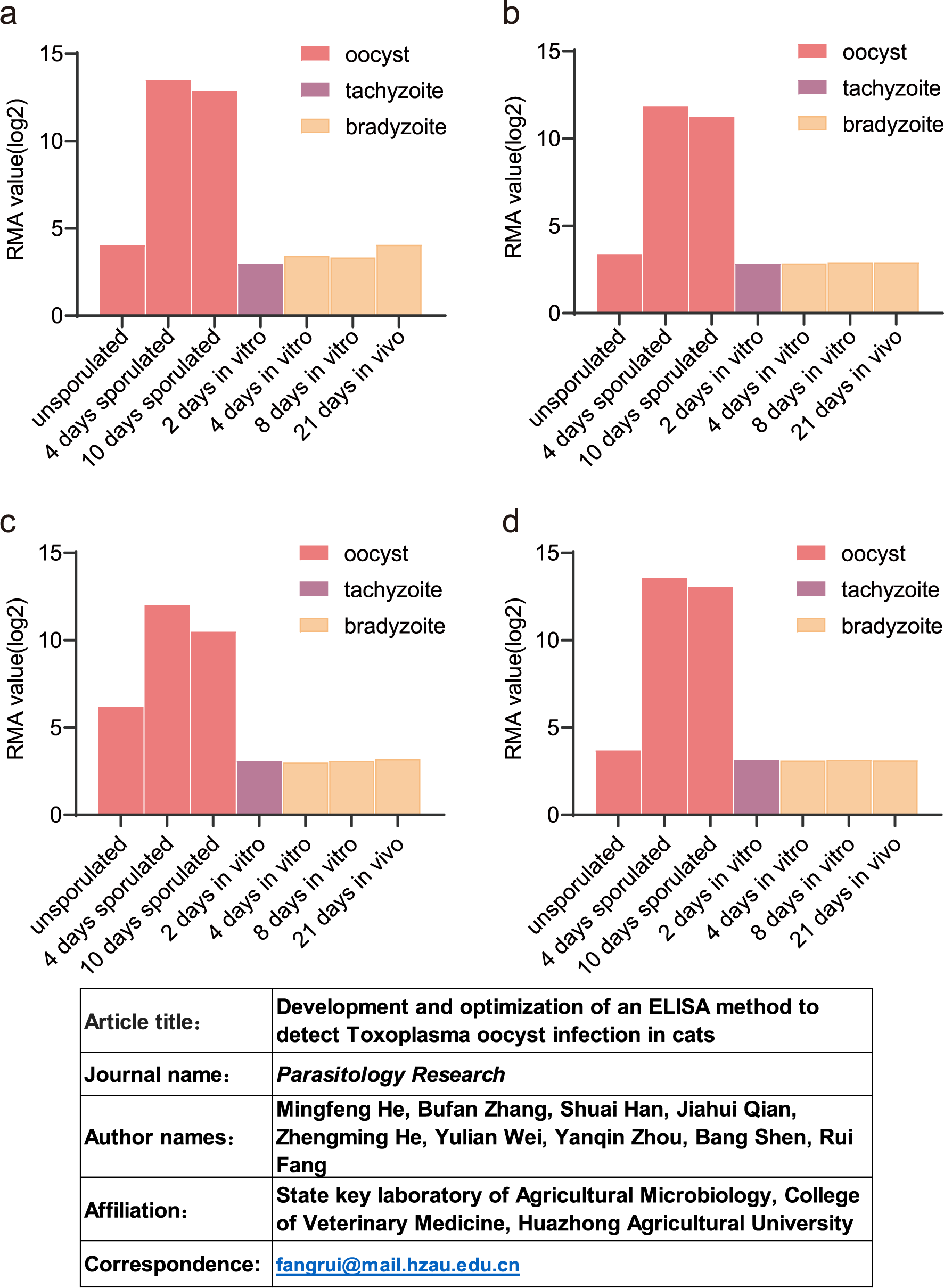

Supplement: Supplementary file 1 — (ZIP 3.47 MB) [file 436_2025_8523_MOESM1_ESM.zip › 974fa741-7d04-4376-b7ca-9c891b6d1dc7-supplementary files/Supplementary Fig. 1.tif]

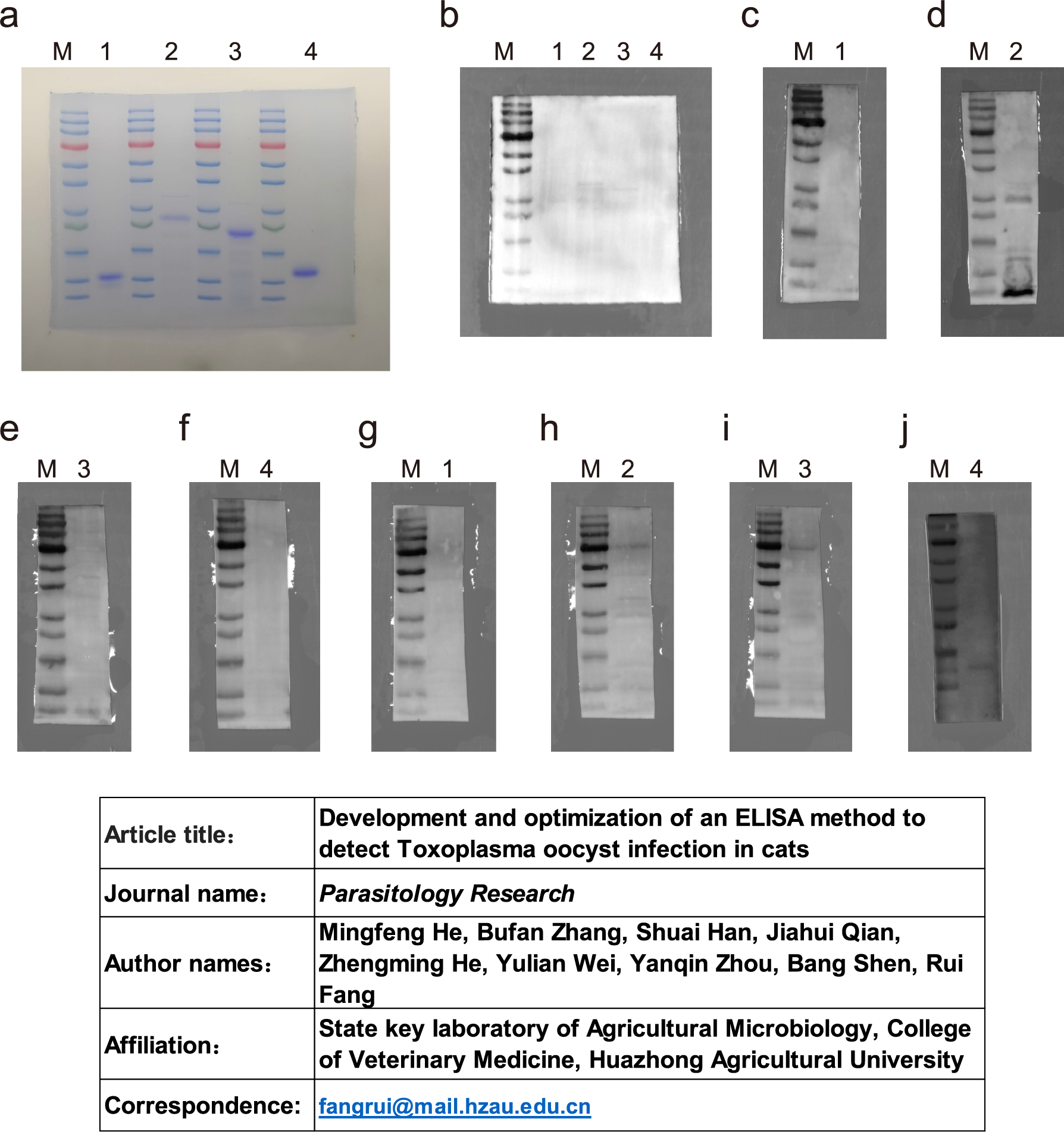

Supplement: Supplementary file 1 — (ZIP 3.47 MB) [file 436_2025_8523_MOESM1_ESM.zip › 974fa741-7d04-4376-b7ca-9c891b6d1dc7-supplementary files/Supplementary Fig. 2.tif]
